# Use of off-label and unlicensed medicines in neonatal intensive care

**DOI:** 10.1371/journal.pone.0204427

**Published:** 2018-09-25

**Authors:** Haline Tereza Matias de Lima Costa, Tatiana Xavier Costa, Rand Randall Martins, Antônio Gouveia Oliveira

**Affiliations:** 1 Integrated Multiprofessional Health Residency Program—Neonatal Intensive Care Unit, Pharmacy Department, Health Sciences Centre, Universidade Federal do Rio Grande do Norte, Natal, RN, Brazil; 2 School Maternity Januário Cicco, Health Sciences Centre, Universidade Federal do Rio Grande Norte, Natal, RN, Brazil; 3 Pharmacy Department, Health Sciences Centre, Universidade Federal do Rio Grande do Norte, Natal, RN, Brazil; Federal University of Sergipe, BRAZIL

## Abstract

**Purpose:**

To evaluate the use of off-label and unlicensed medicines in a neonatal intensive care unit (NICU) of a teaching maternity hospital specialized in high risk pregnancy.

**Methods:**

A prospective cohort study was conducted between August 2015 and July 2016. All newborns admitted to the NICU who had at least one medication prescribed and a hospital stay longer than 24 hours were included. The classification of off-label and unlicensed drugs for the neonatal population was done according to the information of Food and Drug Administration.

**Results:**

A total of 17421 medication items were analyzed in 3935 prescriptions of 220 newborns. The proportion of newborns exposed to off-label drugs was 96.4%, and to unlicensed medicines was 66.8%. About one-half (49.3%) of the medication items were off-label and 24.6% were unlicensed. The main reason for off-label and unlicensed classification was, respectively, frequency of administration and the administration of adaptations of pharmaceutical forms.

**Conclusions:**

Although there are actions to encourage the development of pharmacological studies with neonates, this study observed a high rate of prescription and exposure of newborns to off-label and unlicensed drugs in NICUs and pointed out areas of neonatal therapy that require scientific investment.

## Introduction

Newborns represent a patient population with special concerns regarding drug therapy, because prematurity and low birth weight may have a significant effect on the pharmacokinetics and pharmacodynamics of many drugs, making the administration of medicines rather delicate [1–2]. Newborns can be admitted to a neonatal intensive care unit (NICU) for several reasons, including extreme prematurity or complications from delivery, [3–5] where they receive multiprofessional care and are often administered medicines that do not have proof of safety and efficacy obtained from randomized clinical trials in this patient population [6–7].

Several international organizations have encouraged the conduct of pharmaceutical clinical trials with newborns [6–8] in an attempt to overcome a number of barriers that have been identified, such as ethical issues involving this vulnerable population, low accrual rates because of the small size of this population, small volume of biological samples that can be obtained from neonates, changing pharmacokinetics and pharmacodynamics with the postnatal age, as well as financial considerations [6,7]. Since 1997 the Food and Drug Administration (FDA), first through the adoption of FDA Modernization Act (FDAMA), have been working in pediatric researches [9]. Ten years after, the European started the encouragement through the European (EU) Pediatric Regulation by EU Medicines Agency (EMA) and a document released in October 2017 shows a growth of medicines for children [10].

Despite this effort, the use off-label and unlicensed drugs in neonatology has been repeatedly reported in the literature [4, 5, 11–14]. Off-label drugs are defined as medicines being administered in ages, indications, dosages and routes of administration that are not in conformity with the marketing authorization issued by the country’s regulatory agency [15]. Unlicensed drugs are imported medicines, contraindicated for neonatal use, as well as those that are adapted or manipulated by health care professionals to make the drug appropriated for neonates [16–17].

The incidence of off-label and unlicensed medicines prescribed in NICU varies from 34% to 95.6% and from 5.7% to 34.6%, respectively [4, 5, 12–14, 17–21]. Furthermore, 44% to 100% of all neonates hospitalized at NICUs are administered at least one off-label or one unlicensed medicine [5, 12, 13, 17, 19, 20]. This large variation in published results is mainly due to different study designs, to variations in the clinical settings or in the study populations, as well as on the adopted definition of off-label and unlicensed drugs. For example, some studies considered contraindicated medicines as off-label drugs while others classified them as unlicensed [16, 20–23]. Other studies did not consider medicines contraindicated to neonates [13, 19]. Still others considered parenteral nutrition and intravenous hydration as unlicensed medicines [4, 18]. Two prospective studies defined off-label drugs only in one aspect, the child age [5, 20]. Additionally, two large studies were based on a retrospective analysis of nationwide patient databases [24–25].

Because of the heterogeneity of the published results, there is no clear picture yet of the magnitude of the problem and of the potential risks to which the neonate population is exposed within NICUs. The lack of information about the safety and efficacy of drugs increases the risk of poor clinical outcomes, of adverse drug reactions (ADR) and medication errors when off-label and unlicensed medicines are prescribed to neonates [12, 18, 26]. These risks are increased in this hospitalized population because, at NICUs, there are typically multiple drugs prescribed mostly to preterm babies with low birth weight, and those two characteristics have significant impact on the pharmacokinetics and pharmacodynamics of drugs [18].

Therefore we aimed to evaluate the issue of off-label and unlicensed drug utilization in the setting of a NICU, through a prospective cohort study conducted on consecutive newborns admitted to a NICU of a referral maternity hospital throughout a full 12-months period.

## Material and methods

### Ethical statement

This study was approved by the Institutional Review Board under the number 580.201/2014 and all legal representatives authorized the participation of their children in the study by signing an informed consent form.

### Study design

During a full year, from August 2015 to July 2016, we conducted a prospective cohort study in the NICU of a teaching maternity hospital that has 25 NICU beds, an average annual number of admission of 436 newborns and an annual mortality rate of 15 babies. This maternity is a referral centre for high risk pregnancy, women health, gynaecological surgery, and paediatric cardiac malformations.

During the study period, all neonates admitted to the NICU were evaluated for inclusion. Inclusion criteria were: newborns who were admitted at the NICU for longer than 24 hours, who had at least one drug prescribed during the stay, and whose parents signed an informed consent form. Infants over 28 days of life, newborns with a second admission during the study period, and newborns who were prescribed only with parenteral nutrition, continuous intravenous hydration, oxygen therapy, blood products or electrolytes, were excluded.

From each newborn, data was collected on gender, gestational age (GA), birth weight, APGAR score, admission diagnosis, length of stay, and information on all drugs administered during NICU stay (generic names, indication for use, dosage, frequency and route of administration) by a neonatology pharmacist. The indication for use of the medicines was obtained from detailed patient charts and, when in doubt, the attending physician was consulted. Information about possible or probable ADR was also collected.

All prescribed medicines were classified according to the Anatomical Therapeutic Chemical (ATC) classification system and categorized as labelled, off-label or unlicensed according to the FDA approval criteria, available in the https://www.accessdata.fda.gov/scripts/cder/daf/index.cfm [27]. The drugs were considered unlicensed if: (1) their use was contraindicated for use in neonates, (2) they were extemporaneous preparations that were manufactured or modified by the nurses to make the medicines appropriated to newborns, and (3) they were imported drugs without a marketing authorization in this country [16,17]. Additionally, medicines prescribed in a pharmaceutical form different from the form licensed by the FDA were considered unlicensed. The off-label category included all medicines whose prescription was not in conformity with the marketing authorization issued by the FDA, such as age, indication, dosage and route of administration [15].

### Statistical analysis

Based on the average admission rate at the NICU, we estimated that 220 newborns eligible for the study would be admitted to the NICU during the study period, to which about 12,000 medication items would be prescribed. This sample size would allow the estimation of the percentage of prescribed unlicensed drugs with an accuracy within 0.9 percentage points, and the estimation of the percentage of off-label prescriptions with an accuracy within 0.8 percentage points, with 95% confidence. With a sample of 12,000 medication items we expected, based on the results of a prior pilot study, that 6.600 (55%) would be unlicensed drugs and 3,840 (32%) off-label drugs. Therefore, the maximum error of the estimates obtained in the group of unlicensed drugs would be less than 1.2 percentage points and in the group of off-label drugs would be less than 1.6 percentage points, with 95% confidence. These accuracy levels were considered adequate for the research objectives.

The newborns were categorized according to GA as term (≥ 37 weeks), preterm (between 28 weeks and 36 weeks and 6 days), and extremely preterm (< 28 weeks). Based on the birth weight, the new-borns were categorized in non-low birth weight (>2500 g), low birth weight (< 2500 g), very low birth weight (<1500 g), and extremely low birth weight (< 1000 g).

Descriptive statistics are presented for demographic and clinical data as number and percentage or mean ± standard deviation. Exact binomial 95% confidence intervals (CI) are presented for the proportion of off-label and unlicensed drugs, the proportion of newborns exposed to these drugs, the number of drugs prescribed per neonate, the distribution of these drugs by ATC classification, the dosage forms and the routes of administration used. The statistical analysis was performed using Stata release 11 (Stata Corporation, College Station, TX, USA).

## Results

From August 2015 to July 2016, 308 newborns were eligible for the study. From these, 14 parents refused participation and the parents of 74 newborns could not be located. The final analysis set consisted of 17421 prescribed items in 3935 prescriptions from 220 newborns, of which 101 (46.3%) were females, 43 (19.5%) were term, 134 (60.9%) preterm, and 43 (19.5%) were extremely preterm. The mean gestational age was 32.4 ± 4.4 weeks, (range 23.4 to 42.4 weeks) and the mean NICU stay was 18.3 ± 19.4 days (median = 12 days, range 1 to 106 days). The number of prescriptions and the number of prescribed medicines per patient were, respectively, 17.9 ± 19.2 (median 11, range 1 to 106) and 8.2 ± 6.2 (median 7, range 1 to 33). Of the 17421 items prescribed to the patients, 27.9% (*n* = 4868) were antimicrobials for systemic use, followed by agents acting on the alimentary tract and metabolism (*n* = 4245, 24.4%), nervous system (*n* = 3536, 20.3%*)*, cardiovascular system *(n* = 2324, 13.3%) and respiratory system (*n* = 1369, 7.9%). Thirteen neonates received more than 20 different drugs and two newborns had more than 30 medicines prescribed during the hospitalization. The characteristics of the study population are summarized in **Table** 1.

**Table 1:** Patient caracteristics of the study population.

Variable	Values	
Female sex (n, %)	101	46.3
Gestational age (weeks)	32.4	4.4
Gestational age classification (n, %)		
Extremely preterm	43	19.5
Preterm	134	60.9
Term	43	19.5
Birth weight (g)	1932.7	1127.6
Birth weight classification (n, %)		
Non-low birth weight	54	24.6
Low birth weight	79	35.9
Very low birth weight	50	22.7
Extremely low birth weight	37	16.8
APGAR score		
1 min	6.6	2.4
5 min	8.1	1.4
Cause for NICU admission (n, %)		
Respiratory system	127	57.7
Prematurity	21	9.5
Nervous system	16	7.3
Infectious disease	14	6.4
Other	32	14.5
Prescriptions per patients	17.9	19.2
Prescribed medicines per patient	8.2	6.2
ATC drug prescription class (n, %)		
Antimicrobials for systemic use	4868	27.9
Alimentary tract and metabolism	4245	24.4
Nervous system	3536	20.3
Cardiovascular system	2324	13.3
Other	2448	14.1
Total	17421	100.0

Data are mean ± standard deviation unless otherwise indicated.

Almost half of the medication items prescribed to the newborn were off-label drugs (*n* = 8591, 49.3%, 95% CI 48.6%-50.1%) and the remaining were almost equally divided between unlicensed drugs (*n* = 4278, 24.6%, 95% CI 23.9%-25.2%) and labelled drugs (*n* = 4552, 26.1%, 95% CI 25.5%-26.8%). Of the 220 neonates, almost all of them (212, 96.4%, 95% CI 93.0%-98.4%) were exposed to at least one off-label drug, and about two-thirds (147, 66.8%, 95%CI 60.1%-73.0%) to at least one unlicensed drug. One hundred percent of the extremely preterm and of the extremely low birth weight babies were exposed to off-label medicines (**Table** 2).

**Table 2:** Prevalence of newborns administered off-label and unlicensed medicines in an intensive care unit according to gestational age and birth weight.

					
Variable		n	%	95%CI	
Off label drug prescription		8591	49.3	48.6	50.1
Unlicensed drug prescription (n, %)		4278	24.6	23.9	25.2
Exposed neonates					
	Off label drug	212	96.4	93.0	98.4
	Unlicensed drug	147	66.8	60.1	73.0
Extremely preterm neonates					
	Off label drug	43	100.0	91.8	100.0
	Unlicensed drug	33	76.7	61.4	88.2
Extremely low birth weight neonates					
	Off label drug	37	100.0	90.5	100.0
	Unlicensed drug	28	75.7	58.8	88.8

Only one baby had neither off-label nor unlicensed drugs prescribed, and 140 (63.6%, 95%CI 56.9%-70.0%) of neonates had both medicines prescribed during the NICU stay. The main reasons for the classification of off-label use (**Table** 3) were dosage and frequency of administration not in conformity with the summary of the product characteristics, which were observed in about 40% of off-label prescriptions.

**Table 3:** Reasons for the classification of off-label drug use in newborns in an intensive care unit.

Reasons	Patients		Prescriptions	
	n = 220		n = 17421	
	n	%	N	%
Age	157	71.4	2151	12.3
Indication	156	70.9	1395	8.0
Dose	190	86.4	3310	19.0
Frequency	185	84.1	3435	19.7
Route	56	25.5	1036	5.9

**Table 4** shows the frequency of off-label and unlicensed prescribing within each ATC class. Among the more often used medicines, anti-infectives for systemic use were prescribed as off-label in 79.0% of the cases, drugs acting on the nervous system in 48.0%, on the respiratory system in 95.0% and cardiovascular agents in 53.9%. There was a high prevalence of intravenous administration among off-label drugs, with fentanyl being the most prescribed off-label drug (*n* = 1358), followed by gentamicin (*n* = 1197) and aminophylline (*n* = 1042). Fentanyl was considered off-label because of the patient age, gentamicin because of the selected dose and frequency of administration, and aminophylline because of the indication, being often prescribed for neonatal apnoea.

**Table 4:** Frequency of off-label and unlicensed drug prescription within each ATC category.

ATC category	Off-label		Unlicensed	
	n	%	n	%
Alimentary tract and metabolism	186	4.4	1461	34.4
Blood and blood forming organs	23	4.0	17	3.0
Cardiovascular system	1253	53.9	805	34.6
Dermatological	20	8.4	200	83.7
Systemic hormonal preparations, excl. sex hormones and insulins	98	100.0	0	0.0
Anti-infectives for systemic use	3844	79.0	0	0.0
Anti-neoplastic and immunomodulating agents	4	100.0	0	0.0
Musculo-skeletal system	23	100.0	1	100.0
Nervous system	1697	48.0	1759	49.7
Anti-parasitic products, insecticides and repellents	0	0.0	2	100.0
Respiratory system	1301	95.0	26	1.9
Sensory organs	133	100.0	7	5.3
All other therapeutic products	9	90.0	0	0.0

Among unlicensed drugs, agents acting on the nervous system were the most prescribed medications (*n* = 1759), with caffeine (*n* = 1226) and phenobarbital (*n* = 487) being the most frequent. Adaptations of solid cardiovascular drugs, such as hydrochlorothiazide, spironolactone and furosemide, to liquid formulations accounted for 15.1% of unlicensed medicines. **Table 5** shows in detail the most prescribed medicines and their respective classifications.

**Table 5:** Frequency distribution of medicines prescribed as off-label and unlicensed according to FDA criteria.

Medicine	Off—label		Unlicensed		Prescriptions	
	N	%	n	%	n	%
Fentanyl	1358	15.8			1358	7.8
Caffeine			1226	28.6	1226	7.0
Gentamicin	1197	13.9			1197	6.9
Aminophylline	1042	12.1			1043	6.0
Furosemide	553	6.4	127	3.0	757	4.3
Meropenem	653	7.6			653	3.7
Ampicillin	574	6.7			587	3.4
Phenobarbital			487	11.4	487	2.8
Dobutamine	435	5.1			435	2.5
Cefazolin	383	4.5			383	2.2
Cefepime	345	4.0			345	2.0
Vancomycin	189	2.2			329	1.9
Amikacin	328	3.8			328	1.9
Hydrochlorothiazide			324	7.6	324	1.9
Spironolactone			286	6.7	286	1.6
Other	1534	17.9	1828	42.7	7683	44.1

## Discussion

The present study analysed prospectively the labelling status of drug therapy of newborns hospitalized in a NICU of a Brazilian teaching hospital over a whole year. The results show that during NICU stay nearly all newborns are exposed to off-label medicines and about two-thirds to unlicensed drugs. Off-label prescription of drugs is highly prevalent in NICUs, as well as prescription of unlicensed medications, occurring in about half and one-quarter of the prescriptions, respectively. Fentanyl, gentamicine and aminophylline are the drugs most commonly prescribed as off-label, and caffeine and phenobarbital the most common unlicensed drugs used in NICUs. Adaptations of drug formulations represent a significant proportion of unlicensed use of medicines.

A small number of studies have addressed this issue. A prospective short Italian study conducted for one month in an Italian NICU analysed 88 treatments given to 38 newborns [17] and concluded that 53% were off-label or unlicensed, and that there was no proportional difference of these agents between premature and full-term babies, contradicting the results obtained by another Italian study, which reported that full-term newborns received more off-label drugs than preterms [17, 18].

In another European prospective study, conducted in Ireland during two months and analysing 900 prescriptions from 110 neonates, 76% of neonates received an off-label drug and 47% an unlicensed drug. All extremely prematures received at least one off-label (most prescribed were benzylpenicillin and gentamicin) and unlicensed (most prescribed was caffeine) medicine [19].

A longer research about the use off-label and unlicensed drugs in NICUs was carried out in Germany over 11 months and enrolled 181 neonates who had been prescribed 1978 drugs, finding that 70% of all newborns received at least one off-label or unlicensed drug [5]. Although this study had a significant number of patients and longer duration than the other studies mentioned above, only the licensing status for age was considered. In another large study conducted during six months in two Estonian tertiary care centres, the licensing status of medicines given to newborns hospitalized in NICUs based on 1981 prescriptions to 348 newborns showed that all preterm babies received at least one off-label or unlicensed drug [20].

A Malaysian survey developed in three paediatric units, including a NICU, analyzed the incidence of off-label and unlicensed medication among 194 hospitalised children and risk factors associated with the prescription of these agents [14]. In a eight weeks study, 86 neonates were admitted to the NICU, 90.7% received off-label and 45.3% unlicensed medicines, and these patients had more off-label and unlicensed prescriptions than children hospitalized in a paediatric intensive care unit and a paediatric high dependency unit [14]. A Pakistani study showed that prematurity, number of medicines prescribed, length of stay and gender were risk factors for off-label and unlicensed drugs prescriptions in nursery [28].

During one month, the drug therapy of eight tertiary-level NICUs of southern Italy was studied in 126 neonates receiving 483 prescriptions, showing that 46.6% of prescriptions were off-label and 11.4% unlicensed drugs, that furosemide was the most prescribed off-label drug, and that unlicensed drugs were more often prescribed to preterm babies because caffeine was the most prescribed unlicensed medicine [4]. In Brazil, a retrospective study evaluated the licensing status of medicines given to 192 newborns from the analysis of 3290 prescriptions, of which 95.6% were of off-label drugs and 11.2% unlicensed drugs, with the highest frequency of these medicines in neonates with gestational age less than 28 weeks [13].

Therefore, there is clear evidence that the prescription of off-label and unlicensed medicines to newborns treated in NICUs is a worldwide practice, with about half the medications (37% to 55% according to published studies) prescribed off-label. Estimates of the percentage of prescription of unlicensed drugs are more heterogeneous, with several studies reporting in the range of 10%-12%, while the study conducted in Malaya and our own study reporting about one-third of prescriptions. Exposure of neonates in NICUs to off-label and unlicensed drugs is very high, with estimates of 76% to 91% and of about 45%, respectively. There are several reasons for the variation of estimates. In one study the authors pointed out the existence of huge differences in neonatal drug information between three consulted information sources [20]. Recently, Flint *et al*. showed a large difference between four Dutch NICUs in relation to the prescription of off-label drugs [29]. In this retrospective study, 54% of neonates were exposed to off-label medicines and the prescriptions of cardiovascular and nervous system drugs had greater discrepancy between the NICUs [29].

It is apparent from our cohort, as well as from several published studies, that systemic anti-infectives are the drugs most often involved, with gentamicin being the antimicrobial most used in an off-label manner. This aminoglycoside often composes the first treatment regimen for neonatal sepsis, especially in very low birth weight infants [30]. As this drug is prescribed off-label regarding dosage and frequency of administration, this should be taken as an argument towards the individualization of gentamicin therapy through the development of pharmacokinetic models for neonates, thereby minimizing the risks of renal and internal ear toxicity, [31] as well as of medication errors associated with administration intervals of 24, 36 or 48 hours that are commonly prescribed in neonatology, as was shown in a study [26].

In order to minimize neonatal pain, which is a contributing factor for an increase in morbidity and mortality among neonates, [32,33] the high-alert medication fentanyl was the most prescribed analgesic, and it was the main prescribed off-label drug regarding age in our cohort. The choice of the analgesic varies according to the profile and practice of each NICU [12, 19]. The fact that opioid analgesics are among the most frequently prescribed off-label medications for neonates is disturbing, considering that for the Institute for Safe Medication Practices this pharmacological group belongs to the group of high-alert medicines that, if incorrectly administered, have an increased risk of causing significant damage to the patient, including death [34].

In respect to drugs acting on the respiratory system, aminophylline was the most often used off-label drug. Again, this is a reason for patient safety concerns, because this xanthine has a narrow therapeutic window, frequently causing ADRs, such as tachycardia [35] especially if cardiovascular drugs like dobutamine and dopamine are administered concomitantly.

Regarding unlicensed drugs, we estimate that two-thirds of the neonates are exposed to them and that prematurity influences the use of these agents. Actually, the most often prescribed unlicensed medicine was caffeine, a therapeutic alternative for neonatal apnoea, a disease that affects newborns under 37 weeks of gestation and virtually all extremely premature babies [35]. Caffeine citrate has recently been licensed in Brazil for parenteral and oral administration, but the magistral preparation of caffeine is still widely used because of its lower cost to the public health service.

The conversion of solid to liquid pharmaceutical forms represents about one-sixth of the unlicensed drugs administered in NICUs, and this practice occurs mostly with cardiovascular medicines because of the unavailability in the market of liquid formulations adequate for administration to the neonatal population, a reality observed in several others countries [12, 21, 22, 31]. As a rule, pharmaceutical adaptations for children, commonly performed by nurses from tablets or other solid formulations for adult use, present insufficient data on their chemical and physical stability, and are not prepared in an adequate environment that complies with sanitary regulations. This practice also contributes to inaccuracy of the dose and may even cause obstruction of nasogastric tubes when administered through them [18, 36, 37].

Our study adds up to a small but growing body of literature showing that neonates are exposed to medication hazards because of the lack of evidence on efficacy, safety and dosage of drugs commonly used to treat serious conditions in this population, such as neonatal sepsis or respiratory distress. Certainly, the ideal solution to this problem would be to conduct pharmacokinetic studies and clinical trials in this population, in addition to the production of pharmaceutical forms adequate for newborns. Until that is done, which is rather unlikely, the best measure to improve the safety of newborns in NICUs is to increase the awareness of the health care team to the issue of off-label and unlicensed prescription. The pharmacists may have an important role in this matter, given their knowledge about information sources on chemical and physical stability of active principles and excipients, and on the correct preparation of magistral formulations, in addition to monitoring the drug therapy [38, 39].

The main limitation of this study is to have been conducted at a single centre, where local clinical practices may diverge somewhat from those of other regions and countries. On the other hand, the study has several methodological aspects that contribute to the validity of the estimates, such as the prospective cohort design with follow-up of neonates until NICU discharge, the 12 months enrolment period avoiding possible seasonal trends on prescription, the selection of a widely recognized drug information source, and the large sample size.

In addition, this study presents a much more comprehensive evaluation of the use of off-label medicines by analyzing off-label status due to age, indication, dose, frequency of administration and administration routes, by relating the use of these medicines to gestational age and weight at birth, as well as placing emphasis not only on patient safety when dealing with off-label and unlicensed drugs, but also on the consequences of these medicines by highlighting potential drug-drug interactions and high-alert medicines.

## Conclusions

The use of off-label medicine affects almost all neonates hospitalized in a NICU, regardless of gestational age and birth weight. The unlicensed use, which is not as frequent as off-label use, is still relevant, however, since the use of unlicensed agents increases with prematurity. Antimicrobials for systemic use, drugs acting on the nervous system, as well as respiratory system and cardiovascular drugs, are the medicines most often implicated in off-label use, while unlicensed prescriptions are mainly of the nervous, cardiovascular and alimentary tract classes.

Due to the high number of off-label and unlicensed drugs prescribed to neonates, efforts are still required from pharmaceutical companies and governments to increase investment in clinical trials to prove the safety, efficacy and quality of neonatal pharmacotherapy, including the production of pediatric formulations whenever adaptations of pharmaceutical forms might compromise patient safety.

There is a large area of research by pharmacy professionals, either in clinical research, pharmacokinetic assays, clinical pharmacy or in the field of pharmaceutical technology.

## Supporting information

S1 File Study dataset. (XLSX)
